# Is the 5-port approach necessary in laparoscopic gastrectomy? Comparison of surgical effects of reduced-port laparoscopic gastrectomy and conventional laparoscopic-assisted gastrectomy: A meta-analysis

**DOI:** 10.1097/MD.0000000000022525

**Published:** 2020-10-16

**Authors:** Hao Lai, Zhen Yi, Di Long, Jungang Liu, Haiquan Qin, Xianwei Mo, Huage Zhong, Yuan Lin, Zhao Li

**Affiliations:** a Department of Gastrointestinal Surgery, Guangxi Cancer Hospital, 71 Hedi Road; b Department of Clinical Laboratory, First Affiliated Hospital of Guangxi Medical University; c Department of Gastrointestinal Surgery, Affiliated Wuming Hospital, Yongning Road; d Guangxi Cancer Hospital, 71 Hedi Road, Nanning, Guangxi Autonomous Region, China.

**Keywords:** gastrectomy, laparoscopic, meta-analysis, reduced-port

## Abstract

**Background:**

: Reduced-port surgery, in which fewer ports are used than those in conventional laparoscopic surgery, is becoming increasingly popular for various procedures. However, the application of reduced-port surgery to the gastrectomy field is still underdeveloped. The aim of this study was to use meta-analysis to address the potentially important advantages of this surgical technique.

**Methods:**

: Embase, PubMed, and Cochrane Library databases were systematically reviewed (through October 2019) to identify studies that compared reduced-port (RPLG) and conventional laparoscopic-assisted gastrectomy (CLG) in patients with gastric carcinoma. The endpoints were postoperative time, length of in-hospital stay, blood loss, retrieved lymph nodes, postoperative complications, time to first flatus, and aesthetic outcome.

**Results:**

: A total of 11 studies, which included 1743 patients (907 RPLG and 836 CLG), were ultimately included in this analysis. Better aesthetic results: were obtained with RPLG (risk ratio 1.578; 95%CI, 1.377–1.808; *P* = .000), although length of in-hospital stay (standard mean difference [SMD] −0.106; 95% CI, −0.222 to 0.010; *P* = .074), time to first flatus (SMD −0.006; 95%CI, −0.123 to 0.110; *P* = .913), and perioperative complications (risk ratio 0.255; 95%CI, 0.142–0.369; *P* = .478) were equivalent. However, operative time was significantly longer (SMD 0.301; 95%CI, 0.194–0.409; *P* = .00), blood loss was greater (SMD −0.31; 95%CI, −0.415 to 0.205; *P* = .000), and fewer lymph nodes were harvested (SMD 0.255; 95%CI, 0.142–0.369; *P* = .000) in the RPLG group.

**Conclusions:**

: Our meta-analysis showed that RPLG is as safe as the CLG approach and offers better aesthetic results for patients with gastric carcinoma. However, basing on current evidence, RPLG was not an efficacious surgical alternative to CLG, as operative time was significantly longer, blood loss was greater, and fewer lymph nodes were harvested in the RPLG group. Additional high-powered controlled randomized trials are required, to determine whether RPLG truly offers any advantages; these future studies should particularly focus on pain scores and aesthetic outcomes.

## 1 Introduction

Gastric carcinoma (GC) is one of the most common malignancies, according to current clinical statistics.^[1]^ Laparoscopic radical gastrectomy is an established minimally-invasive procedure for the treatment of gastric carcinoma.^[2]^ Although evidence regarding the oncologic advantages of laparoscopic surgery is still limited, meta-analyses have demonstrated fewer postoperative complications, shorter hospital stays, and faster recovery after laparoscopic than open surgery.^[3]^ Laparoscopy has also been widely used in patients with gastric carcinoma, especially those with advanced gastric carcinoma who require lymphadenectomy^[4]^; however, surgery is still technically challenging, since lymph node dissection is a key step of radical gastrectomy, and is closely associated with patient prognosis.

The magnifying effect of the laparoscope is an advantage for radical gastrectomy. Initially, conventional laparoscopic-assisted gastrectomy (CLG) required a 5–7 cm mini-laparotomy on the epigastrium, for reconstruction after gastrectomy. Recently, several intracorporeal anastomosis techniques have been developed for reconstruction, using the Billroth I-II and the Roux-en-Y procedures. Reduced port laparoscopic-assisted gastrectomy (RPLG) may lead to less postoperative pain and better aesthetic outcomes^[5]^; RPLG also enables surgeons to perform surgery without the need of an assistant, to reduce additional ports, thereby making RPLG more affordable than CLG.^[5]^ However, technical difficulties, including the lack of specialized instruments, limited operating view, and restricted instrument movement, still consist major obstacles, preventing the wide acceptance of RPLG.^[5]^

To date, several studies have described RPLG application in patients with gastric carcinoma,^[6–14]^ but most of these studies include small sample sizes and inconsistent results. For example, some studies have shown no significant differences in operative time between RPLG and CLG procedures,^[6,14]^ while others have revealed significantly shorter operative times of CLG than RPLG.^[10,15]^

Safety and superiority of RPLG are not yet well established. The objective of our meta-analysis was to compare the short-term outcomes of RPLG and CLG, to determine their relative safety and effectiveness.

## 2 Materials and methods

This is a meta-analysis and an IRB approval and written consent are not required.

Search Strategy: We systematically searched the Embase, PubMed, and Cochrane Library electronic databases (up to October 2019). We used Medical Subject Headings (MeSH) and searched using the following words in all possible combinations: “gastrointestinal,” “gastric,” “stomach,” “reduced-port,” “dual-port,” “triple-port,” and “duet port.” We also manually searched the reference lists of all relevant articles. No language or time restrictions were imposed; 2 reviewers (Hao Lai and Huage Zhong) independently extracted data from each study and resolved conflicts by consensus.

Eligibility Criteria: The inclusion criteria for this meta-analysis were the following:

1. this was a randomized controlled trial (RCT) or an RCT with a retrospective design (controlled clinical trial) that compared RPLG with CLG,
2. the surgeon performed RPLG using any endoscopic or laparoscopic instrument, and
3. the studies contained at least one of the following endpoints: postoperative time, length of in-hospital stay, postoperative complications, retrieved lymph nodes, blood loss, and time to first flatus.

The exclusion criteria were the following:

1. case reports, reviews, quasi-randomized trials, and
2. overlapping data.

### 2.1 Data extraction and risk of bias assessment

Two reviewers (Hao Lai and Huage Zhong) independently extracted and critically appraised the data. The reviewers used the Cochrane Handbook for Systematic Reviews of Interventions to assess the risk of bias.^[15]^ The assessment was based on sequence generation, allocation concealment, blinding, incomplete outcome data, selective outcome reporting, and other sources of bias. A third reviewer (Xianwei Mo) organized a consensus meeting to resolve disagreements.

### 2.2 Study quality assessment

Two independent reviewers (Hao Lai and Huage Zhong) assessed the quality of the studies using the Newcastle-Ottawa Quality Assessment Scale.^[16]^ Elements of this scale include selection, comparability, and outcome. A study can be awarded a maximum of one star for each numbered item within the selection and outcome categories; a maximum of 2 stars can be given for comparability. Each study was classified as either low quality (0–5 stars) or high quality (6–9 stars),^[16]^ and the low-quality studies were excluded.

### 2.3 Statistical analysis

The outcome of interest was considered suitable for the analysis, if it met the following criteria:

1. continuous variables (such as length of postoperative in-hospital stay and operative time) were presented as means and standard deviations on the same scale, and
2. identical variables were analyzed by a minimum of 2 studies; 6 outcome variables were chosen for the analysis: postoperative time, length of in-hospital stay, postoperative complications, retrieved lymph nodes, blood loss, and time to first flatus.

We used version 12.0 of the Stata software (Stata Corp, College Station, TX) to analyze the dates, and we used the risk ratio (RR) and either a fixed effects or a random effects model to analyze the dichotomous variables, according to the absence or presence of heterogeneity. We employed the standardized mean difference (SMD) to analyze the continuous variables. We used the Q-based chi-square test and the *I*^2^ statistic to analyze statistical heterogeneity between the studies, and if the *P* value was less than .05, we considered that as a statistically significant heterogeneity among the studies; subgroup analyses were performed according to the types of surgery being compared, for example, reduced-port laparoscopic distal gastrectomy (RP-LDG) vs conventional laparoscopic distal gastrectomy (C-LDG) and reduced-port laparoscopic total gastrectomy (RP-LTG) vs conventional laparoscopic total gastrectomy (C-LTG).

## 3 Results

The entire study selection process for analysis was abided by the guidelines of the Preferred Reporting Items for Systematic Reviews and Meta-Analyses (PRISMA) and the PRISMA-Protocol guidelines.^[17]^ The results were presented as a PRISMA flow diagram with clearly expressed reasons for exclusion and inclusion at each stage. A total of 432 articles that mentioned RPLG and CLG were carefully screened. We screened the full texts, titles, and abstracts, or a combination of these, and removed any duplicate results. One study was excluded for outcomes that were not expressed as the mean and standard deviation.^[18]^ Ultimately, 11 studies were deemed eligible for the final meta-analysis. The quality assessment of these studies is listed in Table 1.

**Table 1:**
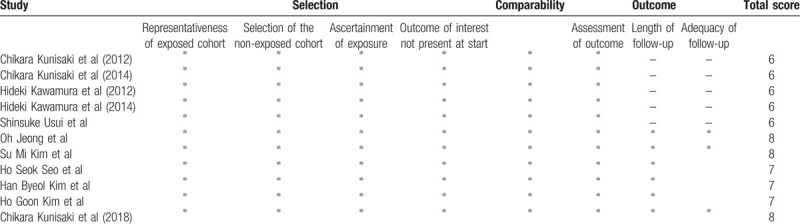
Newcastle-Ottawa Scale of the included studies.

### 3.1 Characteristics of the Included Studies

Table 2 shows the characteristics of the studies included in this meta-analysis; 9 studies were controlled clinical trials and 2 were RCTs. The publication years ranged from 2012 through 2018. A total of 7 trials were reported in Japan, and 5 of them were reported in Korea. A total of 1743 patients (907 RPLG and 836 CLG) were included. The inclusion criteria for patients were described in all studies, and the most similar inclusion criterion for RPLG was preoperative clinical Stage IA (cStage IA) gastric cancer located in the lower and middle part of the stomach. In 2 studies, 2 types of surgery were conducted (laparoscopic distal gastrectomy and laparoscopic total gastrectomy).

**Table 2:**
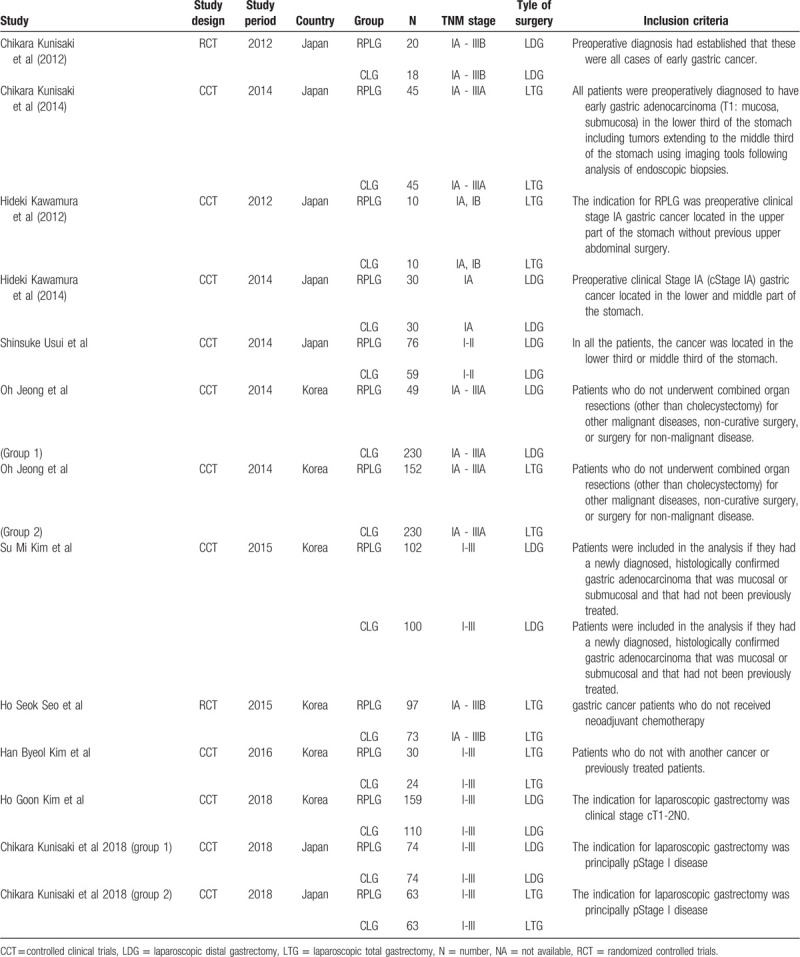
Included studies characteristics.

### 3.2 Outcome measurements

**Figure 1:**
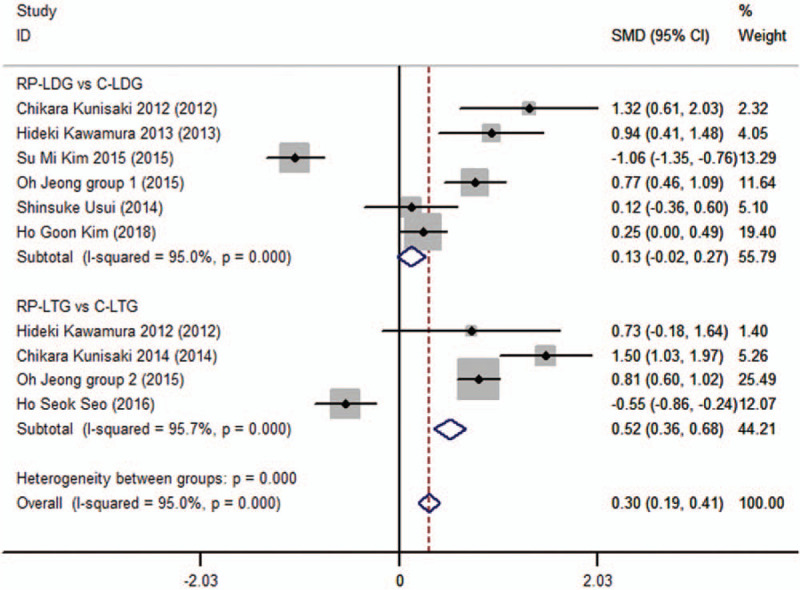
Forest plots of operative time for gastric cancer patients (contrast reduced port laparoscopic-assisted gastrectomy vs conventional laparoscopic-assisted gastrectomy).

Operative times were available for 9 of the included studies.^[6,7,9,10,12–14,19,20]^ Subgroup analysis revealed that the operative time for GC patients was not significantly different between the RP-LDG and the LDG groups (SMD 0.128; 95%CI, −0.015 to 0.272; *P* = .08), with significant heterogeneity (*I*^2^ = 95% and *P*_Q_ = .00 for heterogeneity). Conversely, the operative time was significantly longer in the RP-LTG group than in the C-LTG group of patients with GC (SMD 0.519; 95%CI, 0.357–0.681; *P* = .00), and the overall analysis supported this trend (SMD 0.301; 95%CI, 0.194–0.409; *P* = .00; Fig. 1) with significant heterogeneity (*I*^2^ = 95% and *P*_Q_ = .00 for heterogeneity), but without publication bias (*P* = .67).

**Figure 2:**
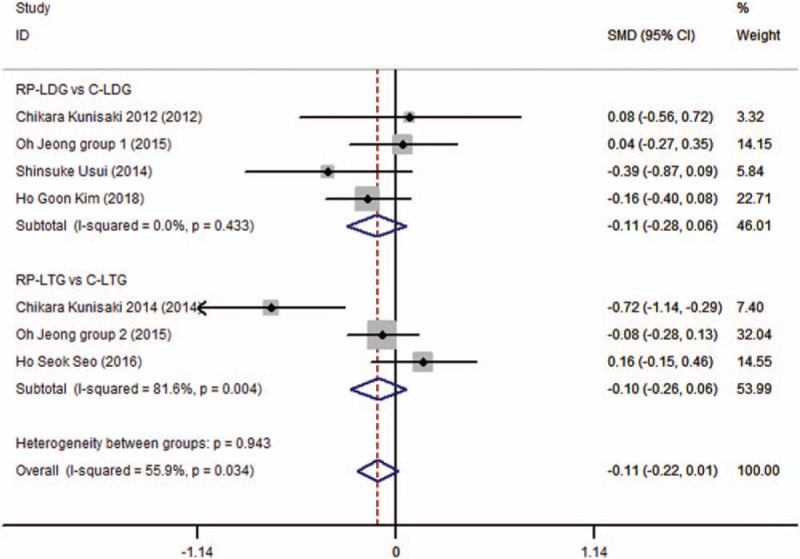
Forest plots of length of hospital stay for gastric cancer patients (contrast reduced port laparoscopic-assisted gastrectomy vs conventional laparoscopic-assisted gastrectomy).

The available data for the length of in-hospital stay was provided by 6 studies, which included 1066 patients.^[6,10,12–14,20]^ In most of these studies, the length of in-hospital stay ranged from 5 to 9 days for both the RPLG and CLG procedures. No significant difference was noted for the length of in-hospital stay between the RP-LDG and C-LDG subgroups (SMD −0.110; 95%CI, −0.281 to 0.061; *P* = .206), or between the RP-LTG and C-LTG subgroups (SMD −0.102; 95%CI, −0.260 to 0.056; *P* = .206). Pooled analysis revealed no significant differences between the RPLG and CLG procedures (SMD −0.106; 95%CI, −0.222 to 0.010; *P* = .074; Fig. 2), with heterogeneity across the trials (*I*^2^ = 55.9% and *P*_Q_ = .034 for heterogeneity) but without publication bias (*P* = .53).

**Figure 3:**
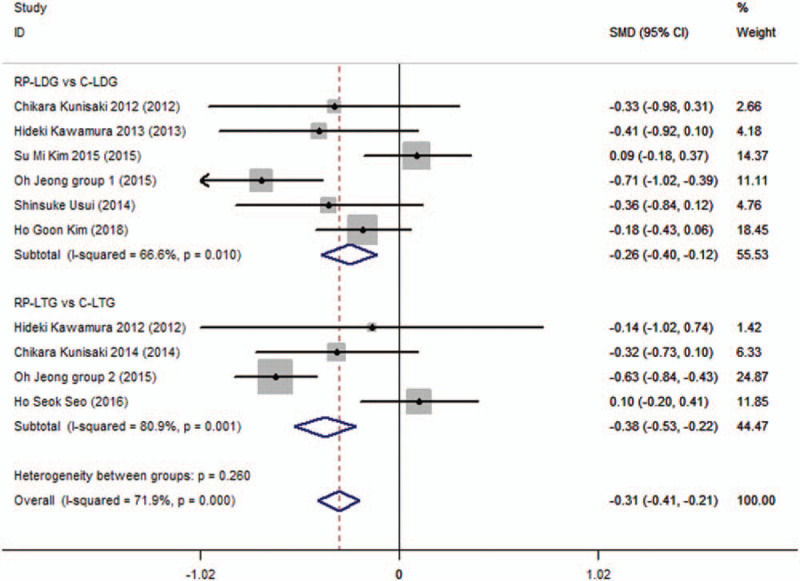
Forest plots of blood loss for gastric cancer patients (contrast reduced port laparoscopic-assisted gastrectomy vs conventional laparoscopic-assisted gastrectomy).

A total of 9 studies have provided available data on blood loss and included 1348 patients.^[6,7,9,10,12–14,19,20]^ Meta-analysis showed statistically significant differences between all the subgroups (SMD −0.31; 95%CI, −0.415 to 0.205; *P* = .000; Fig. 3). The CLG subgroups had significantly lower blood loss, without heterogeneity across trials (*I*^2^ = 71.9% and *P*_Q_ = .000 for heterogeneity), and no publication bias was observed (*P* = .77).

**Figure 4:**
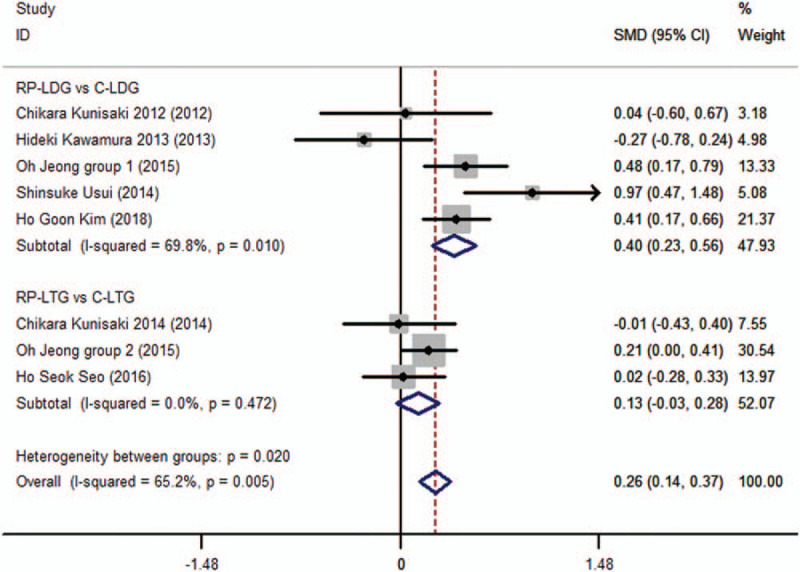
Forest plots of retrieved lymph nodes for gastric cancer patients (contrast reduced port laparoscopic-assisted gastrectomy vs conventional laparoscopic-assisted gastrectomy).

A total of 7 studies, which included 1126 patients, provided available data regarding retrieved lymph nodes^[6,7,10,12–14,20]^, showed statistically significant difference between the RP-LDG and C-LDG subgroups (SMD 0.395; 95%CI, 0.231–0.559; *P* = .000), and no significant difference between the RP-LTG and C-LTG subgroups (SMD 0.126; 95%CI, −0.031 to 0.283; *P* = .117). However, the pooled analysis showed a statistically significant difference between the RPLG and CLG groups (SMD 0.255; 95%CI, 0.142–0.369; *P* = .000; Fig. 4, supplementary information), with heterogeneity across trials (*I*^2^ = 65.2% and *P*_Q_ = .005 for heterogeneity), but with no publication bias (*P* = .81).

**Figure 5:**
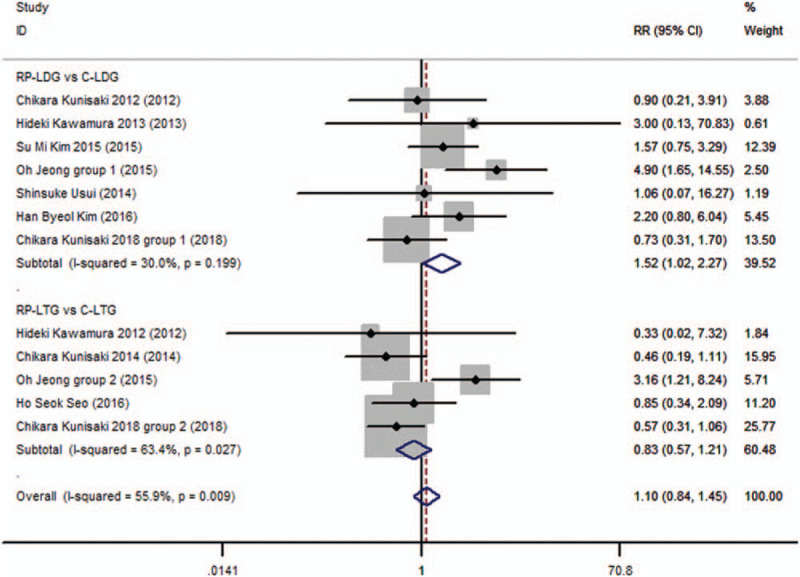
Forest plots of complications for gastric cancer patients (contrast reduced port laparoscopic-assisted gastrectomy vs conventional laparoscopic-assisted gastrectomy).

Complications, such as wound infections, ileus, intestinal obstruction, anastomotic bleeding, intra-abdominal abscess, and lung disease, were found in this meta-analysis. In most of the studies, the most commonly seen complications were wound infection and ileus. Patients with these complications were healed with conservative treatment. Subgroup analysis also revealed that the complications differed significantly between the RP-LDG and C-LDG subgroups (RR 1.520; 95%CI, 1.017–2.271; *P* = .041). Conversely, complications did not show significant differences between the RP-LTG and C-LTG subgroups (RR 0.830; 95%CI, 0.571–1.208; *P* = .331), and the overall analysis supported this trend (RR 0.255; 95%CI, 0.142–0.369; *P* = .478; Fig. 5, supplementary information), with significant heterogeneity (*I*^2^ = 55.9% and *P*_Q_ = .009 for heterogeneity) but without publication bias (*P* = .53).

A total of 6 studies, which included 1058 patients, provided available data for the time to first flatus.^[6,7,10,12,13,19]^ No significant difference was found in the complication rates in any comparative subgroup analysis; the pooled analysis also supported this trend (SMD −0.006; 95%CI, −0.123 to 0.110; *P* = .913), without heterogeneity across trials (*I*^2^ = 41% and *P*_Q_ = .110 for heterogeneity) and without publication bias (*P* = .73).

**Figure 6:**
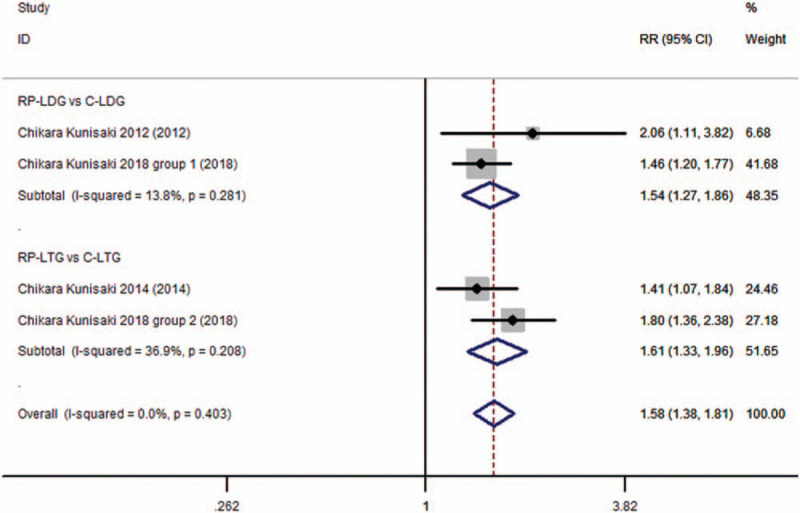
Forest plots of cosmetic effect for gastric cancer patients (contrast reduced port laparoscopic-assisted gastrectomy vs conventional laparoscopic-assisted gastrectomy).

The aesthetic effect was evaluated in 3 studies,^[10–12]^ and meta-analysis revealed a better aesthetic effect in the RDPG subgroup than in the CLG subgroup (RR 1.578; 95%CI, 1.377–1.808; *P* = .000; Fig. 6, supplementary information), without heterogeneity across trials (*I*^2^ = 0.00% and *P*_Q_ = .403 for heterogeneity) and with no publication bias (*P* = .94).

### 3.3 Sensitivity analysis

A sensitivity analysis was conducted to analyze the inclusion criteria of our meta-analysis, to determine whether these criteria would affect the results. Studies included in the meta-analysis were deleted, one study at a time, to determine the influence of each individual dataset on the pooled OR or SMD. The corresponding pooled results were essentially unaltered (data not shown), indicating that our results were statistically sound.

### 3.4 Risk of publication bias

We used Begg funnel plots and Egger regression test to detect the presence of publication bias in our meta-analysis. The confidence interval (CI) and effect estimate was indicated by Begg funnel plots. The publication bias is likely to be minimal for those studies and outcomes if the Egger regression test suggests a distribution of symmetry around the effect evaluations. No publication bias was observed in the outcomes of our meta-analysis (*P* > .05).

## 4 Discussion

Reduced-port gastrectomy can sometimes create conflicts between different surgical instruments and may make precise manipulations more difficult to perform.^[21]^ Therefore, the application of this technique to such a technically complex surgery as laparoscopic gastrectomy may be considered inappropriate. However, our meta-analysis showed that the reduced-port procedure for gastric cancer resulted in acceptable short-term patient outcomes, similar to those achieved following the conventional CLG technique. Aesthetic satisfaction was significantly higher in the reduced-port group than in the CLG group.

Arguments against RPLG often center on whether this procedure may increase operative times produce and costs.^[22]^ Longer operative times mean that patients are exposed to protracted anesthesia, which increases the direct costs, morbidity, and even mortality rates.^[23]^ Our meta-analysis showed that operative times were similar in the RP-LDG and CLDG subgroups; however, significant differences were noted between the RP-LTG and C-LTG subgroups. This perhaps reflects the complexity of the RP-LTG procedure compared to RP-LDG, as RP-LTG is one of the most difficult laparoscopic surgical procedures, and its learning curve for surgeons is longer. Other reasons could include different study designs, sample size, and lack of uniform surgical instrument usage. We believe that surgeons will overcome these limitations and shorten the operative time, as their experience increases.

Our meta-analysis showed that RPLG results in a smaller number of lymph nodes harvested and greater blood loss when compared with CLG. Since reduced-port surgery sometimes creates difficulties in retracting the organ in the right direction with correct retraction power. Optimal organ retraction is important, as it achieves a good operative view and enables the surgeon to perform safe lymph node dissection and intracorporeal anastomosis within an acceptable operative time.^[24]^ We believe that this limitation may also be overcome as surgeons perform a sufficient number of CLG procedures.

In all laparoscopic surgeries, adequate retraction and counter-traction are essential for the control of the orientation of the organs during surgery and for the prevention of intraoperative complications.^[24]^ As already mentioned before, difficulties in manipulating organs in the right direction with appropriate force are sometimes encountered in reduced-port gastrectomy. These technical difficulties can theoretically increase intraoperative damage to the vessels, organs, and surrounding tissues. In our meta-analysis, the most common complications were wound infection and ileus. Patients with these complications were healed with conservative treatment.

The CLG procedure was originally developed in an attempt to further minimize the access site injury, by reducing the number of stab wounds on the abdominal wall. The true value of CLG may be its final aesthetic outcome. In our meta-analysis, only 3 studies provided postoperative aesthetic data,^[10–12]^ and the RPLG procedure showed significantly better aesthetic results for patients. Thus, further studies that use a standard evaluation methodology are needed to verify the true value of CLG, with regard to the aesthetic outcome.

Overall, our meta-analysis showed that RPLG is a feasible and safe procedure, comparable to CLG, despite longer operative time. In addition, RPLG offers a significant advantage in terms of aesthetic outcomes. We believe that surgeons may overcome their learning curves, as their experience with the RPLG procedure increases.

Our meta-analysis has some limitations. Firstly, the most important endpoints for RPLG were pain score and aesthetic outcome; however, most studies have reported only short-term outcomes, with long-term outcomes lacking; secondly, the whole research has some biases, because some of the involved studies had small sample sizes or were retrospective analyses, and only 2 RCTs were included; these may potentially produce bias which affect the result. Furthermore, surgical instrument usage was not uniform, which could also potentially cause bias.

## 5 Conclusions

Our meta-analysis showed that RPLG is as safe as the CLG approach and offers better aesthetic results for patients with GC. However, basing on current evidence, RPLG was not an efficacious surgical alternative to CLG, as operative time was significantly longer, blood loss was greater, and fewer lymph nodes were harvested in the RPLG group. Additional high-powered controlled randomized trials are required, to determine whether RPLG truly offers any advantages; these future studies should particularly focus on pain scores and aesthetic outcomes.

## Author contributions

Zhen Yi, Yuan Lin and Zhao Li conception and design of the study; Jungang Liu, Huage Zhong, Yuan Lin and Haiquan Qin analysis and interpretation of data; Hao Lai drafting the article; Di Long revising it critically for important intellectual content; Xianwei Mo final approve of the version to be submitted.

**Data curation:** Jungang Liu, Haiquan Qin, Huage Zhong, Yuan Lin, Zhao Li.

**Project administration:** Zhen Yi, Yuan Lin.

**Visualization:** Xianwei Mo.

**Writing – original draft:** Hao Lai.

**Writing – review & editing:** Di Long.
